# Vancomycin-Loaded Polycaprolactone Electrospinning Nanofibers Modulate the Airway Interfaces to Restrain Tracheal Stenosis

**DOI:** 10.3389/fbioe.2021.760395

**Published:** 2021-11-18

**Authors:** Yanan Zhao, Chuan Tian, Kunpeng Wu, Xueliang Zhou, Kexing Feng, Zhaonan Li, Zijian Wang, Xinwei Han

**Affiliations:** ^1^ Department of Interventional Radiology, The First Affiliated Hospital of Zhengzhou University, Zhengzhou, China; ^2^ Hubei Province Key Laboratory of Allergy and Immune Related Disease, Department of Biomedical Engineering, Wuhan University School of Basic Medical Sciences, Wuhan, China; ^3^ Ministry of Education Key Laboratory for the Green Preparation and Application of Functional Materials, Hubei University, Wuhan, China; ^4^ Department of Urology, Zhongnan Hospital of Wuhan University, Wuhan, China

**Keywords:** airway stent, polycaprolactone, vancomycin, tracheal stenosis, antibacterial

## Abstract

Site-specific release of therapeutics at the infected trachea remains a great challenge in clinic. This work aimed to develop a series of vancomycin (VA)-loaded polycaprolactone (PCL) composite nanofiber films (PVNF-n, *n* = 0, 1, and 5, respectively) via the electrospinning technique. The physiochemical and biological properties of PVNF-n were evaluated by a series of tests, such as FT-IR, XRD, SEM-EDS, and antibacterial assay. The PVNF-n samples displayed a typical network structure of fibers with random directions. VA was successfully introduced into the PCL nanofibers and could be sustained and released. More importantly, PVNF-5 showed relatively good antibacterial activity against both methicillin-resistant *Staphylococcus aureus* (MRSA) and *Streptococcus pneumoniae* (SPn). Thus, PVNF-5 was covered onto the self-expandable metallic stent and then implanted into a New Zealand rabbit model to repair tracheal stenosis. Compared to a metallic stent, a commercial pellosil matrix–covered stent, and a PVNF-0–covered metallic stent, the PVNF-5–covered airway stent showed reduced granulation tissue thickness, collagen density, α-SMA, CD68, TNF-α, IL-1, and IL-6 expression. In conclusion, this work provides an anti-infection film–covered airway stent that in site restrains tracheal stenosis.

## Introduction

Tracheal stenosis is usually caused by local or systemic inflammation, tuberculosis, and tumor. It compromises the airstream, causing dyspnoea, and is even life-threatening in severe cases (Murgu et al., 2016). Treatment of tracheal stenosis includes medical therapy, surgical resection, and interventional therapy (balloon dilatation, ablation, and stent placement) (Abia-Trujillo et al., 2020). Among these, stent implantation is safe and effective and can immediately relieve obstruction-induced dyspnea (Gompelmann et al., 2013). With the continuous development of interventional techniques and airway stents, stent implantation for tracheal stenosis has become an increasingly familiar option for treatment (Guibert et al., 2020).

Currently, a variety of airway stents, such as the nickel–titanium alloy stent, silicone stent, 3D printing stent, and ^125^I stent, have been applied for tracheal stenosis (Dutau et al., 2015). However, these stents continue to stimulate hyperplasia of the granulation tissue and easily experience tracheal restenosis, and thus cannot meet the clinical needs (Ha et al., 2020). Nouraei et al. found a very significant correlation between *Staphylococcus aureus* and *Pseudomonas aeruginosa* and the formation of the granulation tissue after airway stent implantation and concluded that granulation tissue formation was related to the infection by specific pathogenic microorganisms (Nouraei et al., 2006). Meanwhile, Ost et al. analyzed 195 airway stent–colonized bacteria and showed that airway stent infection was an independent risk factor for subsequent granulation tissue formation (Ost et al., 2012). Moreover, through the follow-up observation of patients who had received airway stent implantation in our hospital at an early stage, it was found that the incidence of tracheal stenosis combined with bacterial infection was as high as 35.7%. Therefore, we speculated that bacterial infection might be an important factor leading to tracheal stenosis (Abbasidezfouli et al., 2009).

For the problem of bacterial infection, we selected vancomycin (VA) as an anti-infective drug. The reasons are as follows: due to the increased bacterial resistance and the airway forming a link with the outside environment, there are often a variety of bacteria such as *Streptococcus pneumoniae*, B *Streptococcus*, *Haemophilus influenzae,* and *Pseudomonas aeruginosa* present in the airway (Noppen et al., 1999). VA, a universal antimicrobial, has no cross-resistance with other antibiotics (Wang et al., 2018). Its antibacterial mechanism works by interfering with a key component of the bacterial cell wall structure to interfere with the synthesis of cell wall peptidoglycan and inhibits the production of phospholipids and polypeptides in the cell wall, thus killing bacteria by inhibiting bacterial growth and reproduction (Fang et al., 2021). However, VA also has some defects, such as no oral absorption. The infusion time should not be less than 1 h. Rapid intravenous infusion can cause skin reactions, and high intravenous infusion concentrations can cause thrombophlebitis (Gould et al., 2009). Therefore, the construction of airway stents to achieve *in situ* anti-infection therapy is a powerful direction to solve the aforementioned problems.

**FIGURE 1:**
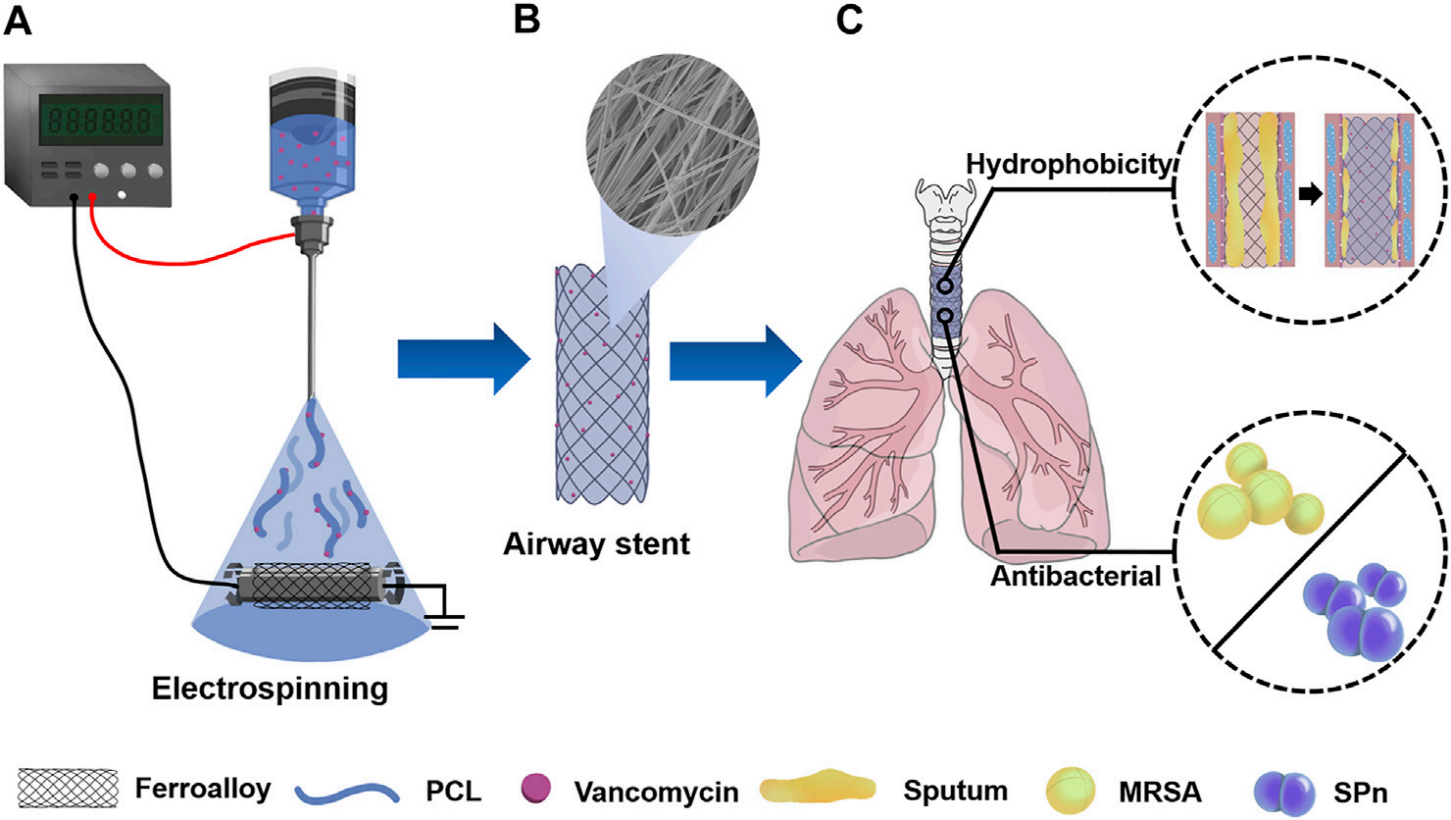
**(A)** PCL/VA film–coated metallic stent (PVNF-n) was prepared by the electrospinning technique. **(B)** PVNF-n samples exhibited an interwoven network structure. **(C)** PVNF-n was transplanted into the trachea to restrain tracheal stenosis, mainly by inhibiting the retention of sputum and the growth of airway bacteria including methicillin-resistant *Staphylococcus aureus* (MRSA) and *Streptococcus pneumoniae* (SPn).

Herein, as shown in Figure 1, we fabricated an anti-infection airway stent PVNF-n consisting of polycaprolactone (PCL) as a high-quality drug carrier and vancomycin (VA) as the antibiotic for drug delivery. The influence of the VA content on the microscopic morphology, hydrophilic/hydrophobic performance, drug release, *in vitro* biocompatibility, and antimicrobial properties was evaluated. Furthermore, *in vivo* tracheal stent implantation was performed to evaluate the safety and efficiency of PVNF-n.

## Methods and Materials

### Materials

Polycaprolactone (PCL, Mw = 60,000) was obtained from SinoBiomaterials Co., Ltd. (Changchun, China). Vancomycin (VA) was purchased from Macklin Biochemical Co., Ltd. (Shanghai, China). Uncovered self-expandable metallic stents (SEMS, 8 × 20 mm) were purchased from MicroTech Co., Ltd. (Nanjing, China). Human embryonic lung fibroblast cells (CCC-HPF-1) were purchased from Shanghai Cell Center (Chinese Academy of Sciences). Methicillin-resistant *Staphylococcus aureus* (MRSA) and *Streptococcus pneumoniae* (SPn) were obtained from Shanghai Institutes for Biological Science (SIBS) and Chinese Academy of Science. Dulbecco’s modified Eagle’s medium, fetal bovine serum, penicillin–streptomycin, and trypsin–EDTA solution were supplied by Sigma-Aldrich Trading Co., Ltd. (Shanghai, China). The Live/Dead Bacterial Viability Kit was sourced from Thermo-Fisher Scientific. Other chemical and biological reagents were obtained from Sinopharm Chemical Reagent Co., Ltd. (Shanghai, China) and used without further treatment.

### Preparation of Nanofiber Film-Covered Airway Stent

10 wt% of the PCL solution was obtained by dissolving 1 g PCL in 9 g hexafluoroisopropanol (HFIP). Then, 0.01 g or 0.05 g of the VA powder was added to the PCL solution to prepare the mixed solution. The obtained solution was used for electrospinning with a voltage of 15 kV and a flow rate of 1.0 ml/h. The distance between the needle tip and SEMS was set as 20 cm. The resultant nanofiber-covered airway stents were coded as PVNF-n (*n* = 0, 1, and 5, respectively), where P refers to PCL, V refers to VA, NF refers to nanofiber film, and n refers to the weight percentage of VA in the nanofiber film, respectively. All products were vacuum-dried for further study.

### Physiochemical Characterizations

Fourier transform infrared spectroscopy (FT-IR, TNZ1-5700, Nicolet, United States) of PVNF-n (*n* = 0, 1, and 5, respectively) was performed in the wavelength range of 4,000–500 cm^−1^. Wide angle X-ray diffraction spectroscopy (WAXD, D-Advance, Bruker, United States) was carried out in the 2θ range of 5–90°. The microstructure of PVNF-n was observed using a scanning electron microscope equipped with an energy-dispersive spectrum analysis system (SEM, JSM-7401F, JEOL, Japan). The water contact angle of PVNF-n was measured by using a contact angle measuring device (First Ten Angstroms, Portsmouth, United States) using the Laplace–Young method. The water droplet was set as 2 µL. The water contact angles were calculated using the computer-interfaced software.

### Drug Releasing Test

The release kinetics of PVNF-n nanofibers was carried out according to a previous report (Kalalinia et al., 2021). Briefly, the PVNF-n samples were incubated in 1 ml phosphate-buffered saline (PBS) in a shaking water bath with a shake speed of 50 rpm at 37°C. At each predefined time point, the PBS solution was completely replaced, and the released VA concentration was measured by mass spectrometry (Thermo TSQ, Shanghai, China).

### CCK-8 Assay

The *in vitro* tests were performed according to ISO10993-12:2007. The PVNF-n samples were sterilized using UV rays and then immersed into DMEM complete culture medium (0.2 g sample per 1 ml culture medium). After being incubated at 37°C for 72 h, the extracts were collected using a 0.22 µm filter. The CCC-HPF-1 cells were digested and then seeded onto 96-well tissue culture plates at a density of 1 × 10^3^ cells per well. After 24 h of incubation, the culture medium was replaced with the extracts. At 1, 2, and 3 d, the original medium was replaced with 100 µL of the fresh medium and 10 µL of the CCK-8 reagent. After further incubation for 2 h, the absorbance value at 450 nm was detected using a microplate analyzer (BioTek, United States).

### Live/Dead Staining Assay

Live/Dead staining assay was performed according to the manufacturer’s protocols for the Live/Dead Viability Kit. Briefly, the CCC-HPF-1 cells at a density of 4 × 10^4^ cells per well were cultured with the extracts of PVNF-n for 1 d. The stocked dye containing 5 µL calcein-AM (4 mM) and 20 µL ethidium homodimer-1 (2 mM) was diluted with 10 ml PBS to prepare the staining solution. The treated cells were incubated with the staining solution for 15 min in a dark place and then rinsed with PBS 3 times. The cell morphology was observed and captured using an inverted fluorescence microscope (Carl Zeiss, Germany). The intensity of live cells (IL) was calculated using the following formula:

$$\text{IL}\left(\%\right)=N_{G}/\left(N_{G}+N_{R}\right)\times 100,$$

where N_G_ and N_R_ represent the average number of live cells and dead cells, respectively.

### Hemolysis Test

This study was performed in compliance with the Animal Care Committee of the First Affiliated Hospital of Zhengzhou University, and in accordance with the National Institute of Health’s guidelines for the care and use of laboratory animals. Female New Zealand rabbits were obtained from the Laboratory Animal Center of Hualan Biological Co., Ltd. (Henan, China). Fresh whole blood from the rabbits was collected and added to an anti-coagulant tube containing 3.8 wt% sodium citrate. Diluted whole blood was prepared by diluting the whole blood with normal saline at a ratio of 1:1.25. 10 µL extracts of PVNF-n was added into a centrifuge tube containing 1 ml normal saline. Distilled water served as the positive control and normal saline served as the negative control. Then, 200 µL diluted whole blood was added into each tube. The aforementioned centrifuge tubes were incubated in a gently shaking water bath for 60 min at 37°C. After centrifugation at 3,000 rpm for 10 min, the supernatant was transferred onto a 96-well tissue culture plate. The absorbance value was measured at 545 nm using a microplate analyzer (BioTek, United States). The hemolysis ratio (HR) was calculated using the following formula (Zhou et al., 2020):

HR(%)=(AS−AN)/(AP−AN)×100,

where AS, AP, and AN represent the average absorbance values of the samples, the positive control, and the negative control, respectively.

### Coagulation Test

Fresh whole blood from the rabbits was centrifuged at 3,000 rpm for 15 min to prepare upper plasma (Shi et al., 2019). The extracts of PVNF-n (*n* = 0, 1, and 5, respectively) was added to plasma and then incubated at 37°C for 10 min. Prothrombin time (PT), activated partial thromboplastin time (APTT), thrombin time (TT), and fibrinogen (FIB) were immediately detected using an automatic coagulation instrument (Leidu, RAC-030). Normal saline served as the control.

### Colony Formation Assay

The antibacterial activity of PVNF-n was tested against two common airway bacteria: methicillin-resistant *Staphylococcus aureus* (MRSA) and *Streptococcus pneumoniae* (SPn). These bacteria were purified and resuspended in 0.85 wt% normal saline for further tests.

PVNF-n was cut into 2 × 2 cm pieces and incubated with MRSA or SPn suspensions (1 × 10^8^ CFU/ml) for 5 h. The obtained bacterial solution concentration was adjusted to 10^−4^–10^−6^ with PBS and then seeded onto Luria-Bertani (LB) dishes and blood agar dishes. After incubation at 37°C overnight, the images of the dishes were captured and the number of bacterial colonies was counted (Wang et al., 2021a). The inhibition rate (IR) was calculated using the following formula:

$$\text{IR}\left(\%\right)=\left(N_{C}-N_{S}\right)/N_{C}\times 100,$$

where N_C_ and N_S_ refer to the average colony number in the blank control and samples, respectively.

### Live/Dead Bacterial Staining Assay

The vitality of bacteria incubated with PVNF-n was assessed by Live/Dead bacterial staining assay. PVNF-n was co-incubated with MRSA or SPn suspensions (1 × 10^8^ CFU/ml) after sterilization for 5 h. The obtained bacterial suspension was stained with DMAO and EthD-III for 15 min at room temperature (Wang et al., 2021a). Afterward, 5 µL of sample was added to a slide and covered with a coverslip. The live and dead bacteria were photographed using a laser confocal microscope (LCS-SP8-STED, Leica, Germany).

### *In vivo* Airway Stent Implantation

This study was performed in compliance with the Animal Care Committee of the First Affiliated Hospital of Zhengzhou University and in accordance with the National Institute of Health’s Guide for the Care and Use of Laboratory Animals. Twenty male New Zealand rabbits weighing approximately 3–3.5 kg were purchased from the Laboratory Animal Center of the Hualan Biological Co., Ltd. (Henan, China). All rabbits were randomly divided into four groups, with five rabbits each in the bare metal airway stent (blank control, BC), commercial airway stent (PC), PVNF-0–covered airway stent, and PVNF-5–covered airway stent groups.

All operations were performed under the guidance of fluoroscopy (Artis zee DSA system, SIMENS, Germany) (Li et al., 2021). First, the rabbits were placed in a supine position with the neck hyperextended in each intervention. Second, a 12-Fr dilator (12 F dilator, Cook Medical, min) was used to dilate an entrance through the rabbit’s mouth. Third, a 5-Fr catheter (Terumo Corporation, Tokyo, Japan) and a 0.035-inch guidewire (Terumo Corporation, Tokyo, Japan) were used to create an intratracheal channel. Finally, a 5-Fr stent delivery system (Terumo Corporation, Tokyo, Japan) using the airway stent was pushed into the intratracheal channel over the guidewire, and then the airway stent was released at least 1.5–2 cm cranially to the carina.

After stenting, 2 ml of blood was collected from the rabbits through the ear vein at the time points of 1, 3, 7, and 14 days, respectively. The aforementioned blood samples were then centrifuged at 4°C 3000 rpm for 15 min to collect the supernatant. Markers of the liver, including alanine aminotransferase (ALT), aspartate aminotransferase (AST), and total bilirubin (TBIL), and markers of the kidney, including blood urea nitrogen (BUN) and serum creatinine (Cr), were detected by an automatic biochemical analyzer (Rayto, China).

Tracheal specimens were obtained by transverse incision of the tracheal stent. After embedding in paraffin, the samples were cut into 5-µm sections and analyzed by hematoxylin–eosin (HE) staining and Masson’s trichrome (MT) staining. Moreover, the immunohistochemistry analyses of α-smooth muscle actin (α-SMA) and CD68 were also performed on the tracheal section, and then the samples were photographed using the fluorescence microscope (IX 73 DP80, Olympus, Japan). The granulation tissue thickness, collagen density, and positive area of α-SMA and CD68 were assessed by Image-Pro Plus software (Media Cybernetics, Silver Spring, MD, United States). Otherwise, the mRNA expression of TNF-α, IL-1, and IL-6 in the tracheal samples was tested by RT-PCR. Additionally, heart, liver, spleen, lung, and kidney samples were also collected and fixed in 10% neutral buffered formalin and examined by HE staining.

### Statistical Analysis

All statistical data were analyzed using SPSS 19.0 software. Experimental data are expressed as mean ± standard deviation. Statistical analysis was performed using one-way analysis of variance (ANOVA), and statistical significance was considered when *p* < 0.05.

## Results

### Constitution of PVNF-n

**FIGURE 2:**
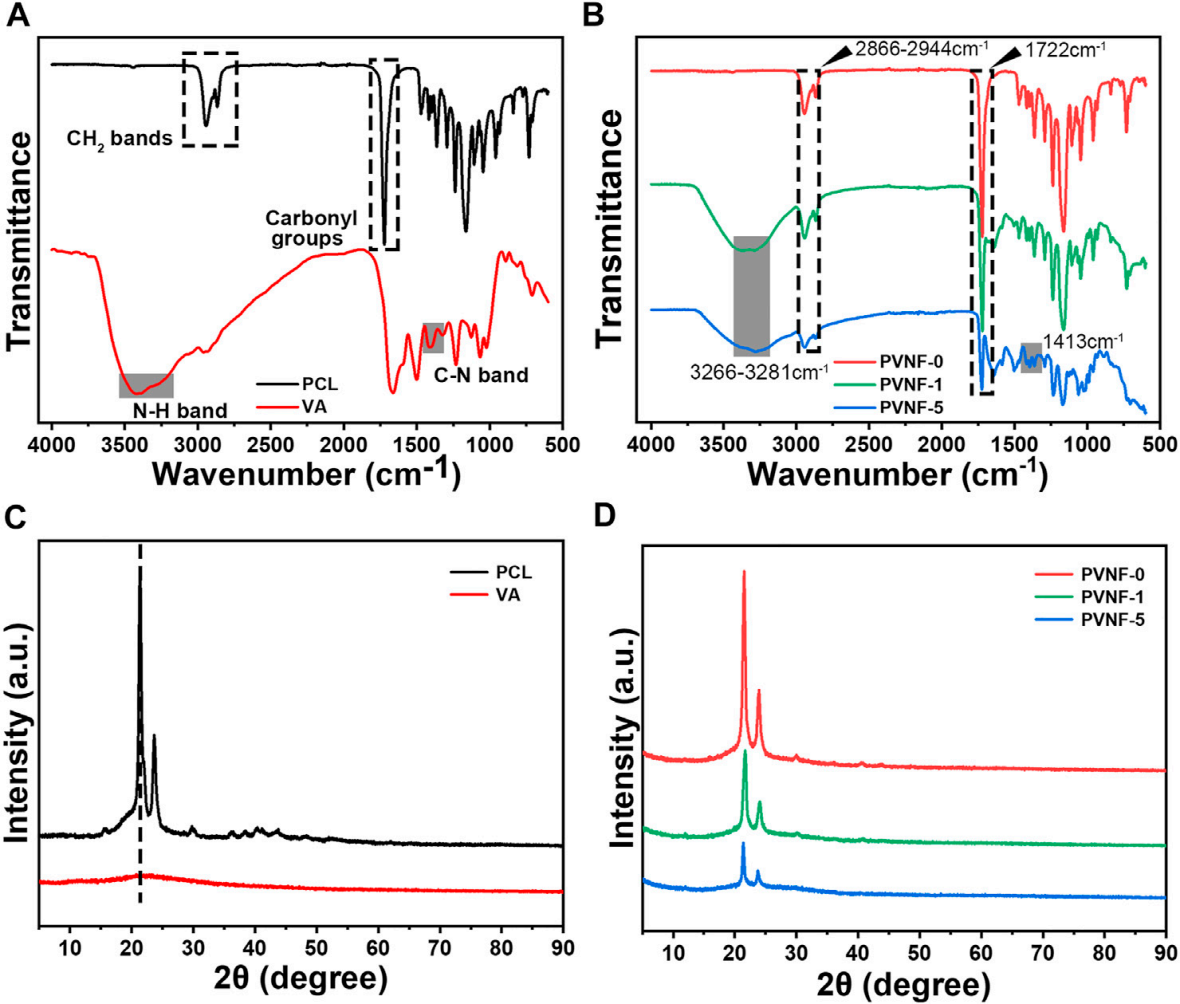
**(A)** FT-IR spectra of the polycaprolactone (PCL) and vancomycin (VA) raw materials. **(B)** FT-IR spectra of PVNF-n (*n* = 0, 1, and 5, respectively). **(C)** XRD spectra of the PCL and VA raw materials. **(D)** XRD spectra of PVNF-n.

FT-IR analysis was performed to identify the chemical component of PVNF-n. As shown in Figure 2A, the FT-IR spectra of the PCL powder exhibited two characteristic peaks corresponding to the asymmetric/symmetric -CH_2_ bands (2944cm^−1^, 2,866 cm^−1^) and carbonyl groups (1720 cm^−1^) (Garrudo et al., 2021). The VA powder exhibited two characteristic peaks corresponding to the stretching of the C–N band (1,413 cm^−1^) and N–H band (3,422 cm^−1^) (Deng et al., 2021). Figure 2B shows the FT-IR spectra of PVNF-n (*n* = 0, 1, and 5, respectively). The characteristic peaks of PCL and VA were obviously presented in PVNF-0, PVNF-1, and PVNF-5, which indicated that VA was successfully introduced into the PCL nanofibers. Furthermore, the XRD spectra results of PCL, VA, and PVNF-n (*n* = 0, 1, and 5, respectively) also proved this statement. As shown in Figure 2C, the PCL powder exhibits two obvious peaks at 21.3° and 23.6° (Golafshan et al., 2020), and no obvious crystal peaks are present in the VA powder. In the XRD peak diagram of PVNF-n, the peak intensity decreased with the increase in the VA content. In conclusion, PVNF-0, PVNF-1, and PVNF-5 were successfully constructed by the electrospinning technique.

### Physicochemical Properties

**FIGURE 3:**
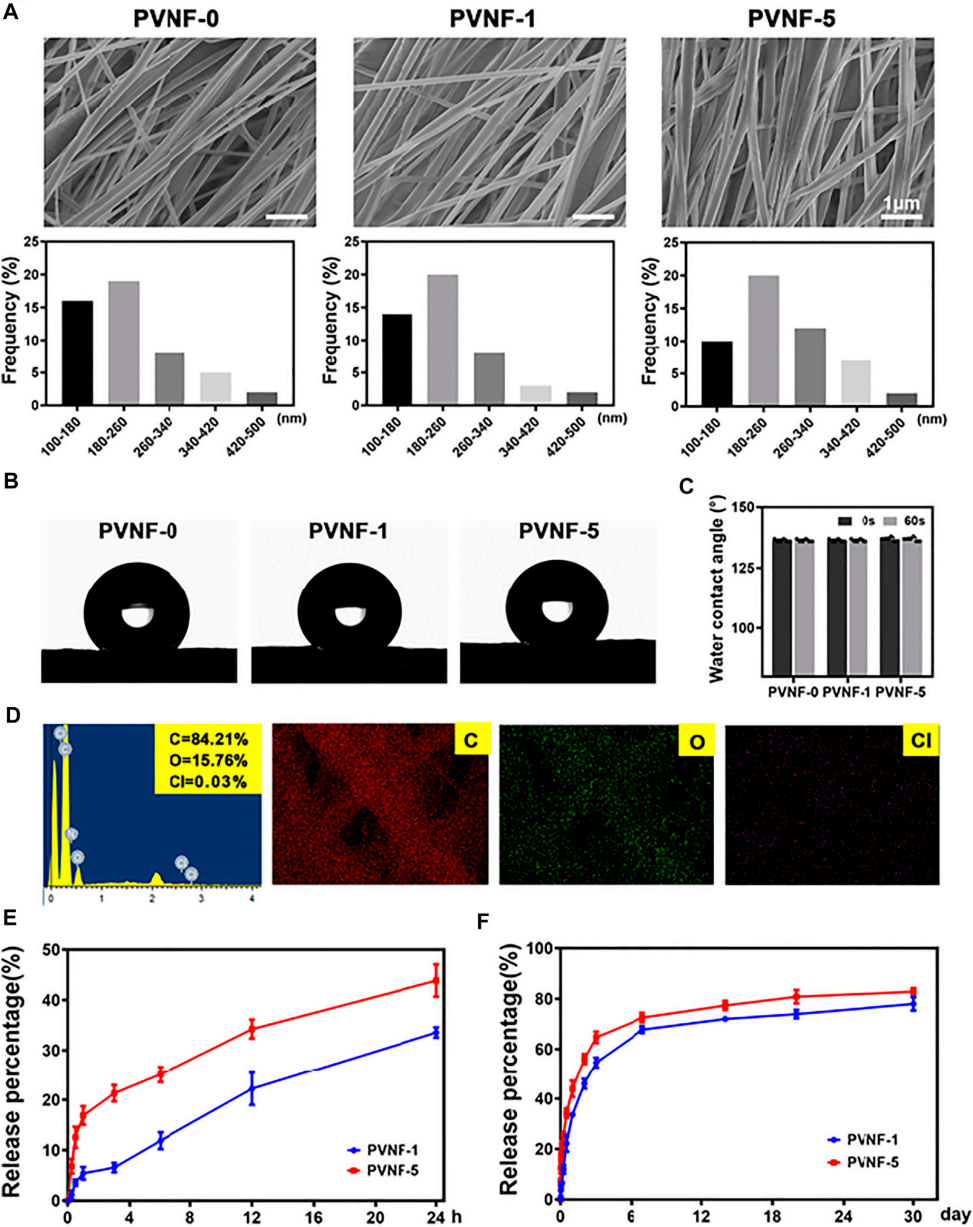
**(A)** SEM images and diameter distribution of PVNF-n (*n* = 0, 1, and 5, respectively). **(B, C)** Representative images and statistical results of the water contact angle (*N* = 5). **(D)** EDS spectrum and elemental mapping of PVNF-5. **(E, F)** Drug release kinetics of the PVNF-1 and PVNF-5 samples (*N* = 5). Scale bar: 1 µm.

SEM images of representative PVNF-n (*n* = 0, 1, and 5, respectively) are provided in Figure 3A. All PVNF-n displayed a network structure of fibers with random directions. The average diameters of PVNF-0, PVNF-1, and PVNF-5 fibers were 212.7 ± 4.7 nm, 207.9 ± 65.4 nm, and 229.0 ± 77.9 nm, respectively. The fiber size varies according to the polymer composition of PVNF-n, which may be related to the concentration of VA. The elements of PVNF-n were tested by EDS. As shown in Figure 3D, the element contents of C, O, and Cl in PVNF-5 were 84.21, 15.76, and 0.03%, respectively. These results further indicated the existence of VA in the PVNF-5 samples.

The water contact angle provides important information about the hydrophilic or hydrophobic characteristics. As shown in Figures 3B–C, the PVNF-0 group exhibited an average water contact angle of 136.55 ± 0.69°, indicating that the surface of PVNF-0 was hydrophobic. With the increase in the VA content, there was no significant change for the water contact angle of PVNF-1 and PVNF-5. The incorporation of VA did not affect the hydrophobicity of PCL.

The release behaviors of PVNF-1 and PVNF-5 were tested in PBS (pH 7.4). As shown in Figure 3E, the release rate of VA increased significantly within 24 h, reaching about 33.6 ± 1.0% for PVNF-1 and 43.9 ± 3.2% for PVNF-5. The rapid release of VA from the stent in the airway has a positive effect on reducing the infective inflammatory response (Wang et al., 2021b). As shown in Figure 3F, with the extension of time, the releasing rate of VA could reach about 78.0 ± 2.6% for PVNF-1 and 82.9 ± 1.4% for PVNF-5 at 30 d. The results suggested that PVNF-n could release VA in the airway for a long time, which played an important role in in-site inhibition of bacterial infection.

### *In vitro* Cytocompatibility Evaluations

**FIGURE 4:**
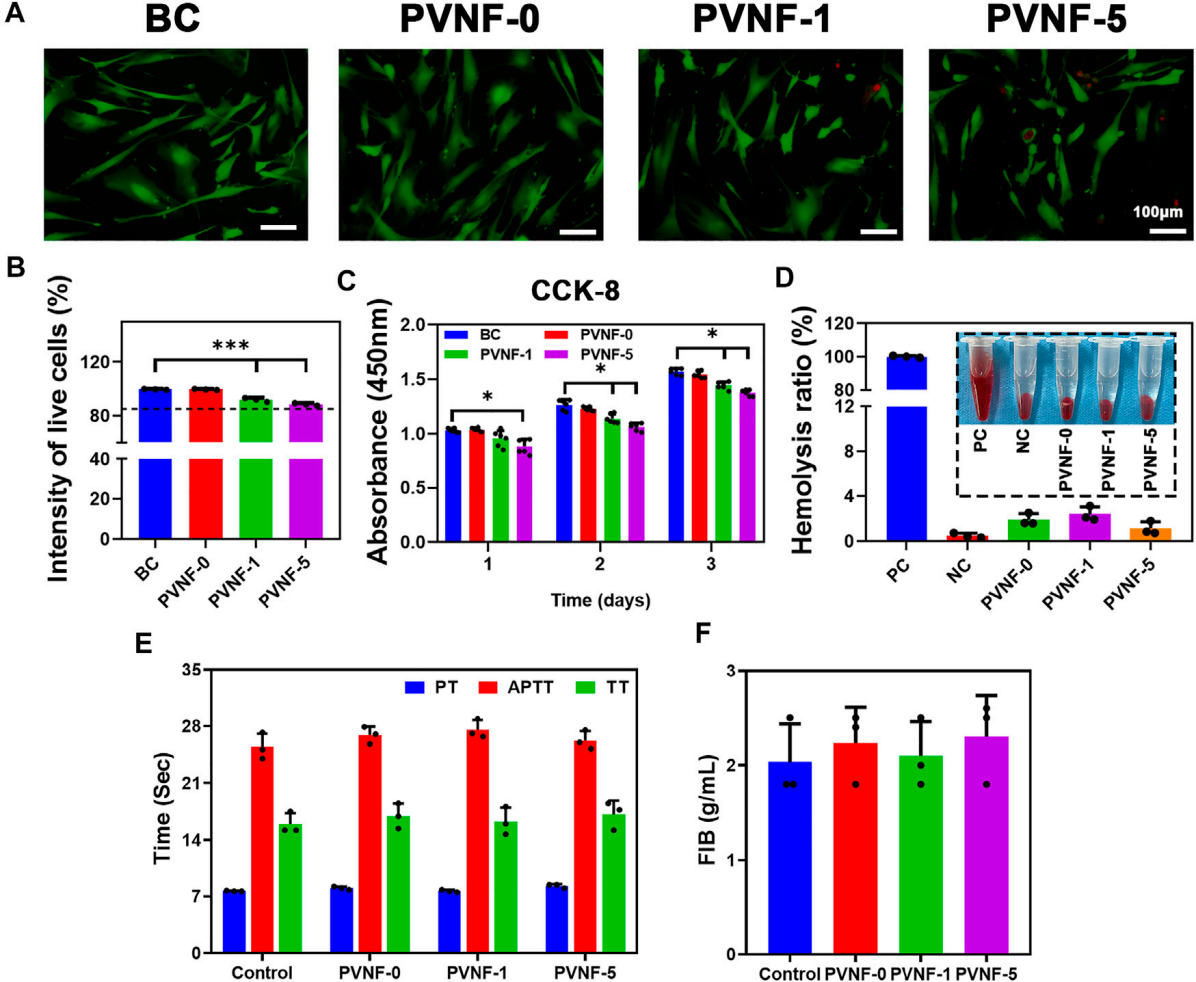
**(A, B)** Live/Dead staining of the CCC-HPF-1 cells and the intensity of live cells (*N* = 3). **(C)** Cell viability of the CCC-HPF-1 cells for 1, 2, and 3 days (*N* = 5). **(D)** Solution of rabbit RBCs treated with the positive control (distilled water, PC), the negative control (normal saline, NC), and PVNF-n (*n* = 0, 1, and 5, respectively), followed by centrifugation. And the hemolysis ratio of rabbit RBCs (*N* = 3). **(E, F)** Prothrombin time (PT), activated partial thromboplastin time (APTT), thrombin time (TT), and fibrinogen (FIB) of PVNF-n (*N* = 3). Scale bar: 100 μm *p < 0.05.

Live/Dead staining and CCK-8 assays were carried out to evaluate the cytocompatibility of PVNF-n samples, and human embryonic lung fibroblast cell (CCC-HPF-1) was used as the model cell. As shown in Figure 4A, green fluorescence represents living cells and red fluorescence represents dead cells. Fluorescence microscopy showed that the majority of CCC-HPF-1 cells were stained in green, and only a few cells were stained in red. In addition, CCC-HPF-1 cells were co-incubated with the extracts of PVNF-n for 1, 2, and 3 days to analyze their proliferation ability. The quantitative results of Live/Dead staining assay are shown in Figure 4B. The intensity of live cells was 99.6 ± 0.4% for PVNF-0, 91.9 ± 1.5% for PVNF-1, and 88.6 ± 1.0% for PVNF-5. As shown in Figure 4C, the OD value of CCC-HPF-1 cells decreased with the addition of VA. The OD value of CCC-HPF-1 cells in PVNF-1 was no significantly different in comparison with that in PVNF-5. In conclusion, PVNF-1 and PVNF-5 have minimal cytotoxicity and can be applied for tracheal stenosis.

The hemolysis rate is an important indicator to evaluate the hemocompatibility. A hemolysis rate less than 5% is considered to have no side effects toward the human body (Lei et al., 2020). The optical photograph of RBCs is showed in Figure 4D. The distilled water group showed obvious hemolysis, while the normal saline group and PVNF-n groups showed no obvious hemolysis. As shown in Figure 4D, the hemolysis ratio of PVNF-n was significantly lower than 5%, indicating that no hemolysis occurred.

The coagulation tests can evaluate the endogenous coagulation system and actively check whether drugs played a role in animals (Herzog et al., 2015). Figures 4E, F shows prothrombin time (PT), activated partial thromboplastin time (APTT), thrombin time (TT), and fibrinogen (FIB) values of PVNF-n. All of the PVNF-n groups were in the normal range compared with the control group, indicating that the PVNF-n samples have no side effect on the endogenous coagulation system.

### *In vitro* Antibacterial Evaluations

**FIGURE 5:**
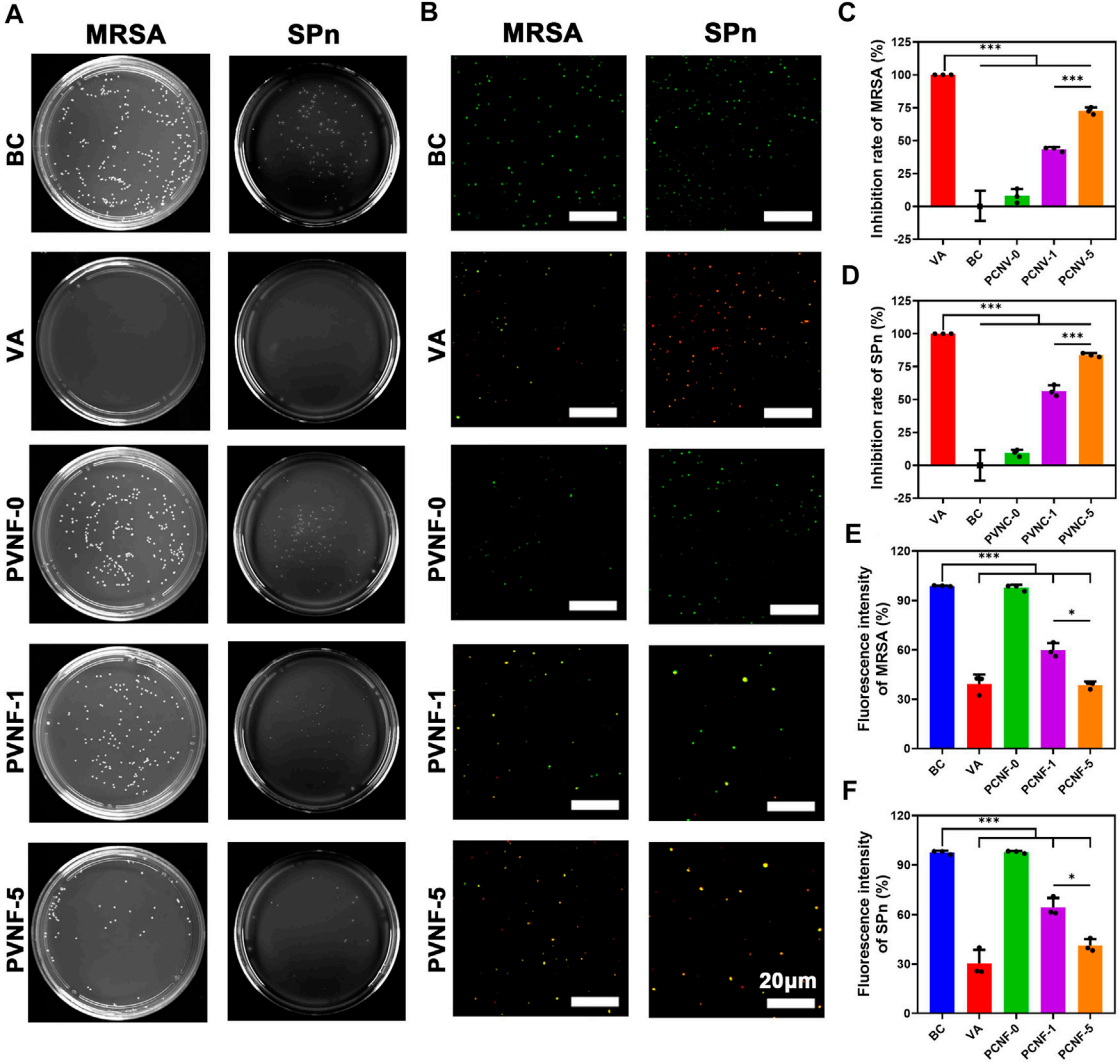
**(A)** Representative images of survival methicillin-resistant *Staphylococcus aureus* (MRSA) and *Streptococcus pneumoniae* (SPn) on LB/blood agar dishes after being treated with PVNF-n (*n* = 0, 1, and 5, respectively). **(B)** Fluorescence microscopy images of DMAO and EthD-III–stained MRSA and SPn incubated in the presence of the medium (blank control, BC), VA (positive control, VA), and PVNF-n (*n* = 0, 1, and 5, respectively) conditioned medium. Live bacteria are stained green and dead bacteria are stained red. **(C, D)** Inhibition rate of PVNF-n for MRSA and SPn, respectively (*N* = 3). **(E, F)** Fluorescence intensity of PVNF-n for MRSA and SPn, respectively (*N* = 3). Scale bar: 20 μm *p < 0.05, and ***p < 0.001.

When the airway is narrow, a variety of bacteria accumulate in the trachea to cause infection (Hillel et al., 2019). It is vital to endow airway stents with enhanced antibacterial activity. In this work, methicillin-resistant *Staphylococcus aureus* (MRSA) and S*treptococcus pneumoniae* (SPn) were used to evaluate the antibacterial performance of PVNF-n. Figure 5A displays the bacterial colony number per field, demonstrating that the antibacterial property of PVNF-n increased along with the increase in the VA content. The quantitative results of the colony number are shown in Figure 5C, D. The inhibition rates (IRs) of MRSA were 7.9 ± 5.3% for PVNF-0, 43.4 ± 1.7% for PVNF-1, and 72.6 ± 2.6% for PVNF-5. The inhibition rates (IRs) of SPn were 9.3 ± 2.5% for PVNF-0, 58.6 ± 4.2% for PVNF-1, and 83.8 ± 1.4% for PVNF-5. Furthermore, the Live/Dead bacterial staining assay was performed. As shown in Figure 5B, the living MRSA/SPn were dyed green, and the dead MRSA/SPn were dyed red. A large number of live bacteria were observed in the blank control, while few live bacteria were observed in the PVNF-5 group. As shown in Figures 5E, F, the fluorescence intensity of live bacteria (MRSA and SPn) in the PVNF-5 group was significantly lower than that in the blank group and PVNF-1 group. In conclusion, PVNF-5 could significantly inhibit bacterial growth and endow the airway stent with antibacterial property to repair tracheal stenosis.

### PVNF-5 Inhibits Tracheal Stenosis in Rabbit Models

**FIGURE 6:**
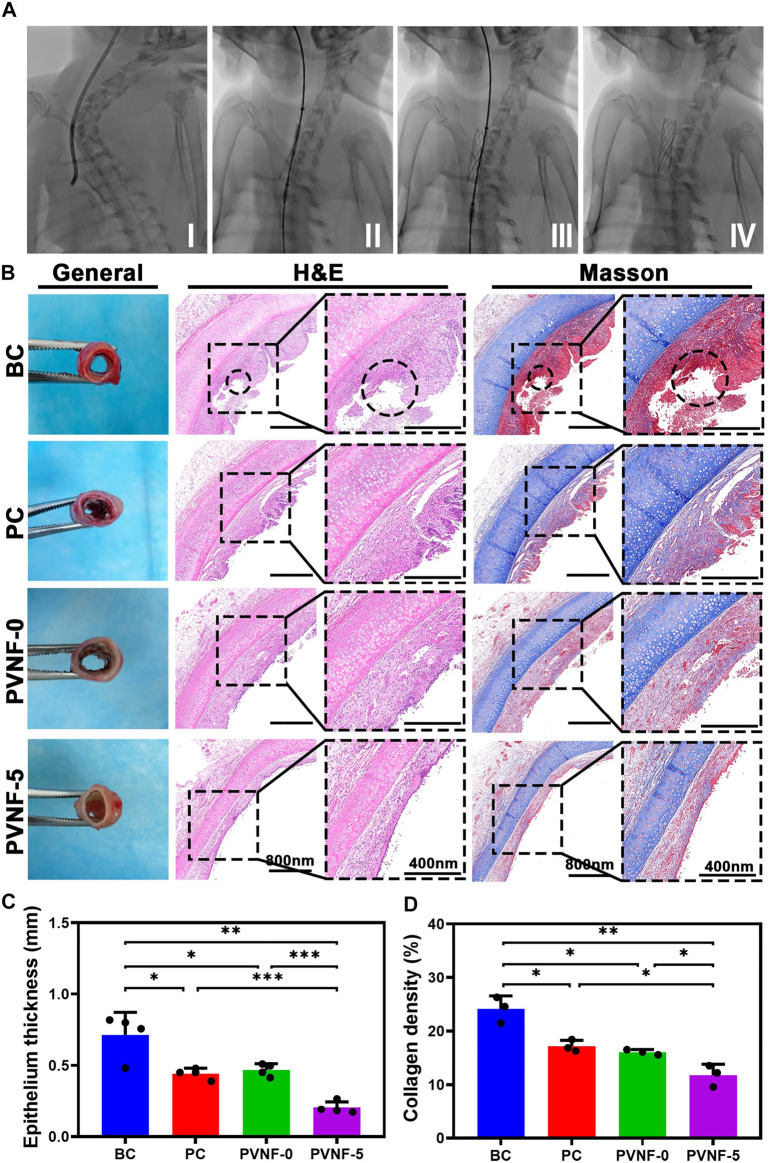
**(A)** Surgical view of stent implantation. **(B)** General observation, HE staining and Masson’s staining in the bare metallic stent (blank control, BC), commercial pellosil matrix–covered stent (PC), PVNF-0–covered metallic stent, and PVNF-5–covered metallic stent. Circles represent the formation of ulcers. **(C, D)** Quantitative results of granulation tissue thickness (*N* = 4) and collagen density (*N* = 3). Scale bar: 400 μm *p < 0.05, **p < 0.01, and ***p < 0.001.

Figure 6A shows the surgical process of transplanting the airway stent into the trachea via an interventional method. After implantation for 1 month, tracheal samples were collected and analyzed by histological methods. As shown in Figure 6B, the general image of the trachea in the BC group presented redness and swelling, while surface edema and related inflammatory manifestations of the intact trachea in the PC and PVNF-5 groups were relatively mild. Further H E and Masson’s staining of tracheal specimens showed that the granulation tissue in the BC group was significantly proliferated and was accompanied by the formation of ulcers (circles in Figure 6B). In addition, the PC and PVNF-0 groups also had different degrees of granulation tissue hyperplasia and epithelial metaplasia. In the PVNF-0 group, the formation of the granulation tissue was less, but the morphology of the mucosal epithelium on the tracheal surface partially disappeared. The statistical analysis shown in Figure 6C revealed that the granulation tissue thickness was significantly reduced in the PVNF-5 group (203.2 ± 42.6 µm), compared to the BC group (714.4 ± 157.4 µm) and even to the PC group (443.1 ± 38.1 µm). Collagen acts as the main component of the extracellular matrix (ECM), and excessive collagen deposition can cause tracheal restenosis (Su et al., 2021). Masson’s staining results indicated that the collagen density was 24.1 ± 2.4% for the BC group, 17.2 ± 1.1% for the PC group, 16.1 ± 0.5% for the PVNF-0 group, and 11.8 ± 2.0% for the PVNF-5 group (Figure 6D). Compared with the other groups, the collagen density was markedly depressed in the PVNF-5 group, suggesting that PVNF-5 could significantly inhibit the collagen deposition.

**FIGURE 7:**
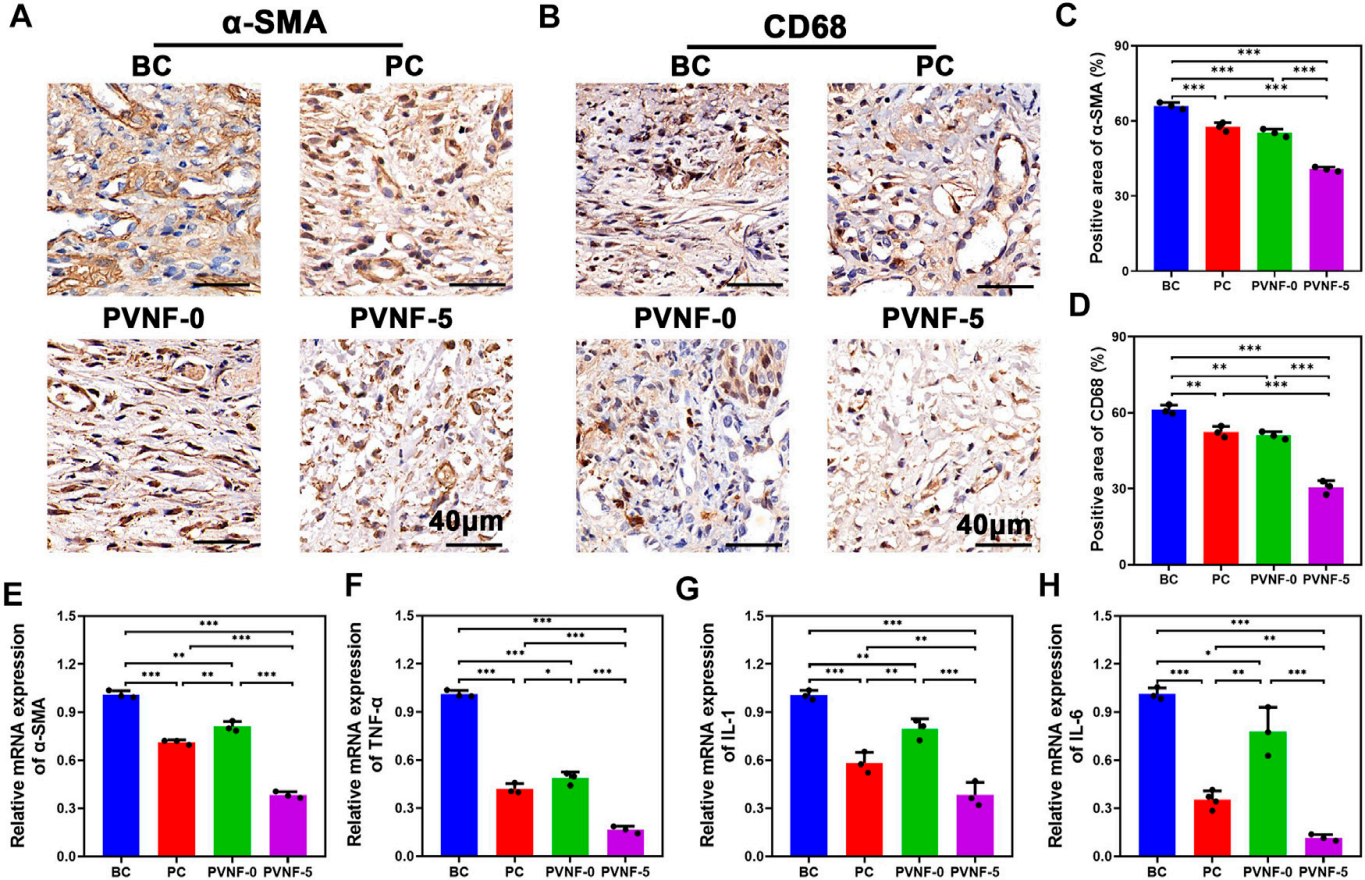
**(A, B)** α-SMA and CD68 immunohistochemical (IHC) staining in the bare metallic stent (blank control, BC), commercial pellosil matrix–covered stent (PC), PVNF-0–covered metallic stent, and PVNF-5–covered metallic stent. **(C, D)** Quantitative results of the positively stained area of α-SMA and CD68 (*N* = 3). **(E, H)** Relative mRNA expression of α-SMA, TNF-α, IL-1, and IL-6, respectively (*N* = 3). Scale bar: 100 μm *p < 0.05, **p < 0.01, and ***p < 0.001.

Furthermore, immunohistochemical (IHC) staining of the tracheal segments for α-SMA was also performed (Figure 7A). α-SMA is a marker of activated fibrogenic cells and myofibroblasts, which are regarded as important effector cells of tissue fibrogenesis (Angelini et al., 2020). As shown in Figure 7C, the positive area of α-SMA in the PVNF-5 group was significantly decreased in comparison with other groups. Figure 7E shows that the mRNA expression of α-SMA in the PVNF-5 group was remarkably lower than that in the other groups, which is consistent with the aforementioned result.

To investigate its anti-infective effects, IHC staining of the tracheal segments for CD68 was also performed (Figure 7B). CD68 is a protein located in the granules of macrophages and is an index of inflammatory response (Chakraborty et al., 2021). As shown in Figure 7D, the positive area of CD68 in the PVNF-5 group was the least, indicating that VA could be released from the composite films and play an anti-infection role. TNF-α, IL-1, and IL-6 are common cytokines used to examine inflammation (Wu et al., 2021). As shown in Figure 7F–H, the mRNA expression of TNF-α, IL-1, and IL-6 was significantly lower in the PVNF-5 group, thereby inhibiting granulation tissue hyperplasia. In summary, the PVNF-5 film–coated airway stent could reduce granulation tissue thickness, collagen density, and the expression of α-SMA, CD68, TNF-α, IL-1, and IL-6, showing that it can be an effective alternative to inhibit tracheal stenosis *in vivo*.

### Safety Analysis of PVNF-n

**FIGURE 8:**
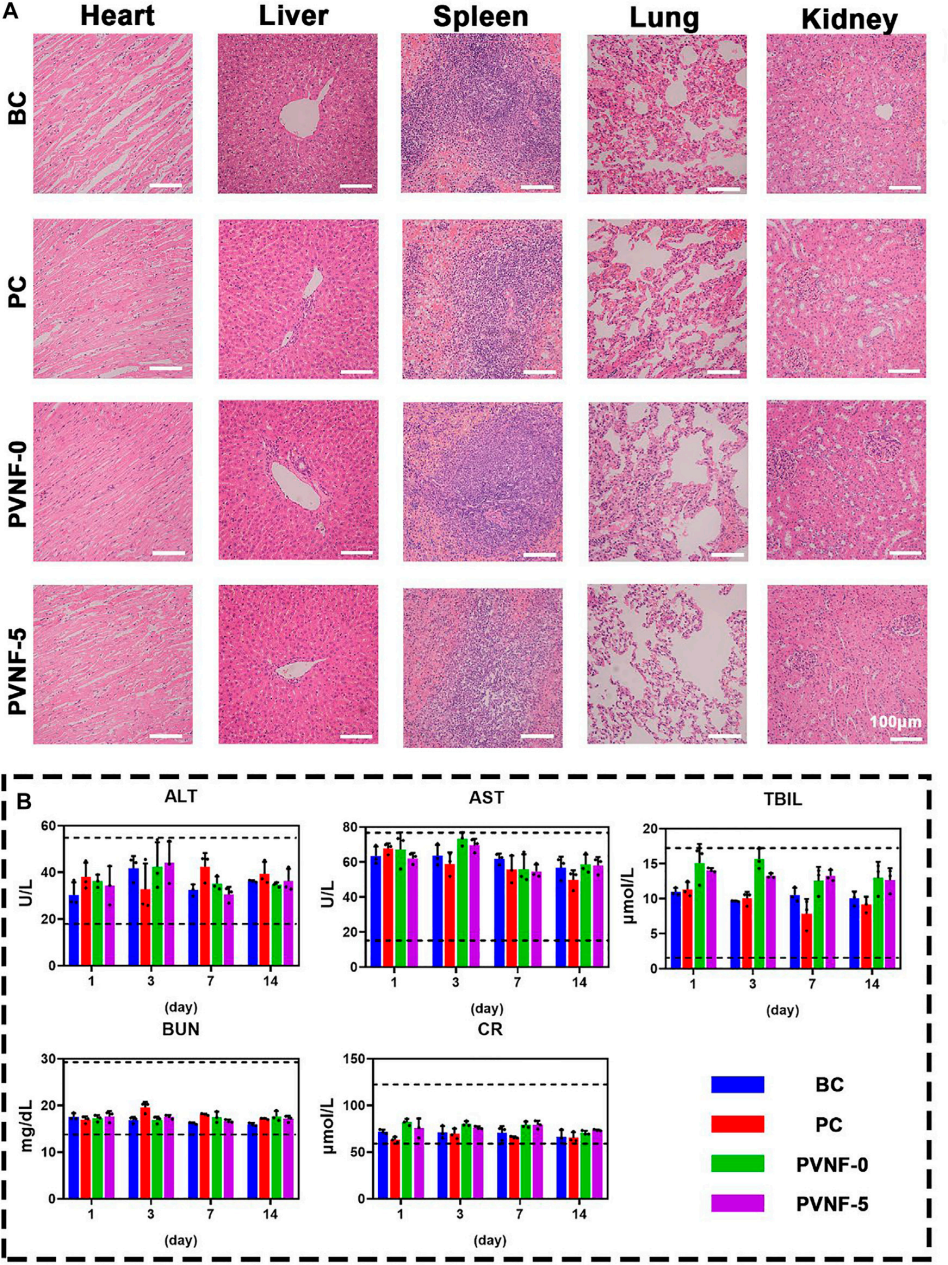
**(A)** Histological evaluations of the toxicity and side effects of the airway stent in the BC, PC, PVNF-0, and PVNF-5 groups, respectively. HE staining of the heart, liver, spleen, lungs, and kidneys was performed. **(B)** Levels of alanine aminotransferase (ALT), aspartate aminotransferase (AST), total bilirubin (TBIL), blood urea nitrogen (BUN), and serum creatinine (Cr) in four groups were detected after stenting at 1, 3, 7, and 14 days (*N* = 3); Scale bar: 100 µm.

The safety of PVNF-n is an important indicator to evaluate its potential application in clinic (Zhang et al., 2020). The HE staining of the heart, liver, spleen, lungs, and kidneys in the BC, PC, PVNF-0, and PVNF-5 groups are shown in Figure 8A, respectively. There were varying degrees of RBC exudation in the lung tissue of the BC, PC, and PVNF-0 groups. In addition, the lung tissue of the BC group and PVNF-0 group exuded fluid, and some tissues consolidated. Compared with that of the other groups, the PVNF-5 group showed the healthiest histological morphology of the lung tissue. Moreover, no obvious pathological changes were observed in the heart, liver, spleen, or kidneys of the BC, PC, PVNF-0, and PVNF-5 groups. The results of liver and kidney function examination, including ALT, AST, BUN, CR, and TBIL, further confirmed good biosafety of PVNF-5 (Figure 6B).

## Discussion

With the development of interventional technology, airway stents have been widely applied in respiratory diseases (Guibert et al., 2020; Paunović et al., 2021). Self-expanding airway stents, such as metallic stents, can quickly expand the narrow airway and alleviate symptoms. However, there is a certain foreign body reaction between the interfaces of stent material and airway tissue, which will inevitably lead to hypertrophic scar and restenosis. Currently, the mechanism of scar hyperplasia has been preliminarily revealed (Mascharak et al., 2021). Chronic airway inflammation caused by pathogen infection is an important driving factor (Roodenburg et al., 2021). Clinical studies also showed that there was a significant correlation between the incidence rate of tracheal stenosis and airway infection (Ost et al., 2012; Grosu et al., 2013). Local application of antibiotics to control pathogen infection is an ideal approach of postoperative management. However, due to the abuse of antibiotics, developing a sensitive antibiotic and advanced drug delivery strategy remains an obstacle toward clinical application.

To solve the problem of tracheal stenosis in commercial metallic stents, a series of VA-loaded PCL electrospinning nanofiber films (PVNF-n) were developed and then coated onto metallic stents to enhance their biocompatibility and antibacterial activity. VA is a powerful antibacterial drug, and its mechanism is to inhibit the synthesis of the bacterial cell wall (Abdul-Aziz et al., 2020). Compared with traditional antibiotics, VA exhibits better killing effect against drug-resistant bacteria (Foster, 2019). At present, there are no reports on VA resistance. In this work, VA and PCL were blended to obtain the composite nanofibers. VA could be sustained and released from the PCL substrate to kill bacteria. For adapting to clinical needs, it is necessary to access and optimize the VA-releasing performance of the products. Compared with the PVNF-1 group, the PVNF-5 group released VA faster in the first 24 h and had higher peak concentration, which was conducive to the rapid removal of pathogens after stent implantation. Based on the results of antibacterial tests *in vitro*, we preferred PVNF-5 for *in vivo* animal experiments. It was found that PVNF-5 could restrain tracheal stenosis. Compared with commercial self-expanding metallic stents, the PVNF-5 film–coated metallic stent could effectively inhibit collagen production and inflammatory infiltration. We concluded that the antibacterial activity of VA played a key role.

Except for the curative effect, the biocompatibility of biomaterials is another decisive factor for their clinical transformation (Chen et al., 2021; Hou et al., 2021). In this work, we systematically evaluated the biocompatibility of PVNF-n film–coated metallic stents by a variety of experimental methods. It was found that PVNF-n neither inhibited the proliferation ability of CCC-HPF-1 cells significantly nor induced obvious hemolysis and coagulation dysfunction. In order to evaluate the long-term biocompatibility of PVNF-n after implantation *in vivo*, we collected the organs and blood samples of treated animals and carried out a series of clinical, biochemical, and pathological tests. The results showed that there were no obvious toxicological changes among the treated animals. PCL is a highly biocompatible polymer, which has been approved by the FDA for application in cardiovascular stents, surgical suture, etc. PCL is one of the best substrates of electrospinning nanofibers. The toxicity of VA is dose-dependent. In this work, low-dose VA (only 1–5%) was adopted and then slowly infiltrated into the surrounding tissues through sustained releasing. While killing local bacteria, the systemic toxicity of VA is reduced as much as possible.

In conclusion, the PVNF-n film–coated metallic stents developed in this work have good clinical curative effect and biocompatibility and initially have the potential for clinical transformation. At present, the approval of medical devices is subject to strict rules, and novel biomaterials need to be tested unprecedented in the transformation research stage. Compared with the existing advanced interventional materials and the processing techniques, we concluded that the products of this work still have some aspects that can be further improved, including controllable regulation of biodegradability, sequential release of dual antibiotics, and realization of combined bioactivities. In the future, we will use the coaxial electrospinning technique to develop the next generation of the antibiotic-loaded film-coated airway stent.

## Conclusion

In this study, a series of PCL/VA fiber film–coated airway stents (PVNF-ns) were successfully fabricated with PCL and VA by electrospinning. The properties of PVNF-n were tested by FT-IR, XRD, SEM-EDS, water contact angle testing, drug release tests, etc. The results showed that PVNF-n possesses a network structure and hydrophobic performance and can sustain the release of VA. PVNF-n also has excellent antibacterial activity against methicillin-resistant *Staphylococcus aureus* (MRSA) and *Streptococcus pneumoniae* (SPn). Furthermore, the results of *in vivo* tests indicated that PVNF-5 could not only reduce granulation tissue thickness and collagen density but also downregulate the expression of α-SMA and CD68. The mRNA expression of inflammatory factors such as TNF-α, IL-1, and IL-6 was inhibited in the PVNF-5 group. In conclusion, this study provides an antibacterial PCL/VA fiber film–coated airway stent to inhibit granulation tissue hyperplasia.

## Data Availability

The original contributions presented in the study are included in the article/Supplementary Material; further inquiries can be directed to the corresponding authors.
